# Structural Heart Alterations in Brugada Syndrome: Is it Really a Channelopathy? A Systematic Review

**DOI:** 10.3390/jcm11154406

**Published:** 2022-07-28

**Authors:** Antonio Oliva, Simone Grassi, Vilma Pinchi, Francesca Cazzato, Mónica Coll, Mireia Alcalde, Marta Vallverdú-Prats, Alexandra Perez-Serra, Estefanía Martínez-Barrios, Sergi Cesar, Anna Iglesias, José Cruzalegui, Clara Hernández, Victoria Fiol, Elena Arbelo, Nuria Díez-Escuté, Vincenzo Arena, Josep Brugada, Georgia Sarquella-Brugada, Ramon Brugada, Oscar Campuzano

**Affiliations:** 1 Department of Health Surveillance and Bioethics, Section of Legal Medicine, Fondazione Policlinico A. Gemelli IRCCS, Università Cattolica del Sacro Cuore, 00168 Rome, Italy; antonio.oliva@unicatt.it (A.O.); francescacazzato993@gmail.com (F.C.); 2 Department of Health Sciences, Section of Forensic Medical Sciences, University of Florence, Largo Brambilla 3, 50134 Florence, Italy; simone.grassi@unicatt.it (S.G.); vilma.pinchi@unifi.it (V.P.); 3 Cardiovascular Genetics Center, University of Girona-IDIBGI, 17190 Girona, Spain; mcoll@gencardio.com (M.C.); malcalde@gencardio.com (M.A.); mvallverdu@gencardio.com (M.V.-P.); aperez@idibgi.org (A.P.-S.); annai@brugada.org (A.I.); 4 Centro de Investigación Biomédica en Red. Enfermedades Cardiovasculares (CIBERCV), 28029 Madrid, Spain; earbelo@clinic.cat (E.A.); jbrugada@clinic.cat (J.B.); 5 Pediatric Arrhythmias, Inherited Cardiac Diseases and Sudden Death Unit, Cardiology Department, Sant Joan de Déu Hospital de Barcelona, 08950 Barcelona, Spain; estefania.martinez@sjd.es (E.M.-B.); sergi.cesar@gmail.com (S.C.); josecarlos.cruzalegui@sjd.es (J.C.); clara.hernandez@sjd.es (C.H.); jvfiolramis@gmail.com (V.F.); georgia@brugada.org (G.S.-B.); 6 European Reference Network for Rare, Low Prevalence and Complex Diseases of the Heart (ERN GUARD-Heart), 1105 AZ Amsterdam, The Netherlands; 7 Arrítmies Pediàtriques, Cardiologia Genètica i Mort Sobtada, Malalties Cardiovasculars en el Desenvolupament, Institut de Recerca Sant Joan de Déu, Esplugues de Llobregat, 08950 Barcelona, Spain; 8 Arrhythmias Unit, Hospital Clinic, University of Barcelona-IDIBAPS, 08036 Barcelona, Spain; nuria.andrews@gmail.com; 9 Institute of Pathological Anatomy, School of Medicine, Catholic University, 00168 Rome, Italy; vincenzo.arena@policlinicogemelli.it; 10 Medical Science Department, School of Medicine, University of Girona, 17003 Girona, Spain; 11 Cardiology Service, Hospital Josep Trueta, University of Girona, 17007 Girona, Spain

**Keywords:** sudden cardiac death, Brugada syndrome, histopathology, forensic pathology, endomyocardial biopsy

## Abstract

Brugada syndrome (BrS) is classified as an inherited cardiac channelopathy attributed to dysfunctional ion channels and/or associated proteins in cardiomyocytes rather than to structural heart alterations. However, hearts of some BrS patients exhibit slight histologic abnormalities, suggesting that BrS could be a phenotypic variant of arrhythmogenic cardiomyopathy. We performed a systematic review of the literature following Preferred Reporting Items for Systematic Reviews and Meta-Analyses Statement (PRISMA) criteria. Our comprehensive analysis of structural findings did not reveal enough definitive evidence for reclassification of BrS as a cardiomyopathy. The collection and comprehensive analysis of new cases with a definitive BrS diagnosis are needed to clarify whether some of these structural features may have key roles in the pathophysiological pathways associated with malignant arrhythmogenic episodes.

## 1. Introduction

Brugada syndrome (BrS) is an inherited cardiac syndrome associated with the increased risk of ventricular tachycardia, ventricular fibrillation (VF), and sudden cardiac death (SCD) in a structurally normal heart. On an electrocardiogram (ECG), the diagnosis of BrS is based on “*ST-segment elevation with type 1 morphology ≥2 mm in one or more leads among the right precordial leads V1 and/or V2 positioned in the second, third, or fourth intercostal space, occurring either spontaneously or after provocative drug test with intravenous administration of sodium channel blockers”* [1]. The type 1 ECG pattern described above is the only one diagnostic of BrS, whereas other repolarization patterns (type 2 and type 3) found in more than one right precordial lead should be considered suggestive of the disease and require further confirmatory investigations. Other known causes of ST-segment elevation in the right precordial leads (phenocopies) must be excluded. BrS is traditionally classified as an inherited cardiac channelopathy because it is associated with ion channel dysfunction or the altered expression/function of proteins associated with ion channels in ventricular cardiomyocytes. It is characterized by incomplete penetrance and variable expressivity. A comprehensive genetic test can identify ~35% of diagnosed BrS patients and covers more than 20 potential genes encoding mainly ion channel components and associated proteins but also structural proteins. The sodium channel protein type 5 subunit alpha (*SCN5A*) gene, in particular, shows deleterious alterations in 30% of diagnosed patients. As the pathophysiological mechanism and functional effects of variants in other genes is still to be clarified, current guidelines recommend genetic analysis of *SCN5A* alone in patients with a BrS ECG [2,3,4].

BrS was first reported in 1992 and was classified as purely of electrical origin; since then, structural cardiac abnormalities have been identified in hearts of some patients with BrS [5,6,7,8]. For instance, right ventricular (RV) enlargement, reduced RV function, larger RV end-diastolic and end-systolic volumes, and left ventricular (LV) midfall late gadolinium enhancement (LGE) are apparent by cardiovascular magnetic resonance (CMR) imaging. LGE may be an early marker of an underlying cardiomyopathy in patients who do not fulfill all the current BrS diagnostic criteria [9,10]. BrS and arrhythmogenic cardiomyopathy (ACM) frequently show overlapping clinical and histopathological features and represent a highly challenging differential diagnosis, thus, leading to a high risk of misdiagnosis when ill-defined features are found [11,12,13]. Commonalities in clinical/histopathologic features and pathophysiological pathways (disorders of the connexome) between BrS and ACM prompted a hypothesis that BrS could be a phenotypic variant of ACM [14,15,16,17,18]; however, this hypothesis remains to be thoroughly tested.

In this review, given these findings and the commonalities between BrS and heart diseases of structural origin, such as ACM, we sought to evaluate if the pathological classification of BrS as a pure channelopathy remains appropriate. To achieve this, we performed a comprehensive review of the topic focusing on the reported macroscopic and microscopic structural alterations in BrS, observed in explanted hearts, autopsies, and endomyocardial biopsies. 

## 2. Material and Methods 

**Figure 1:**
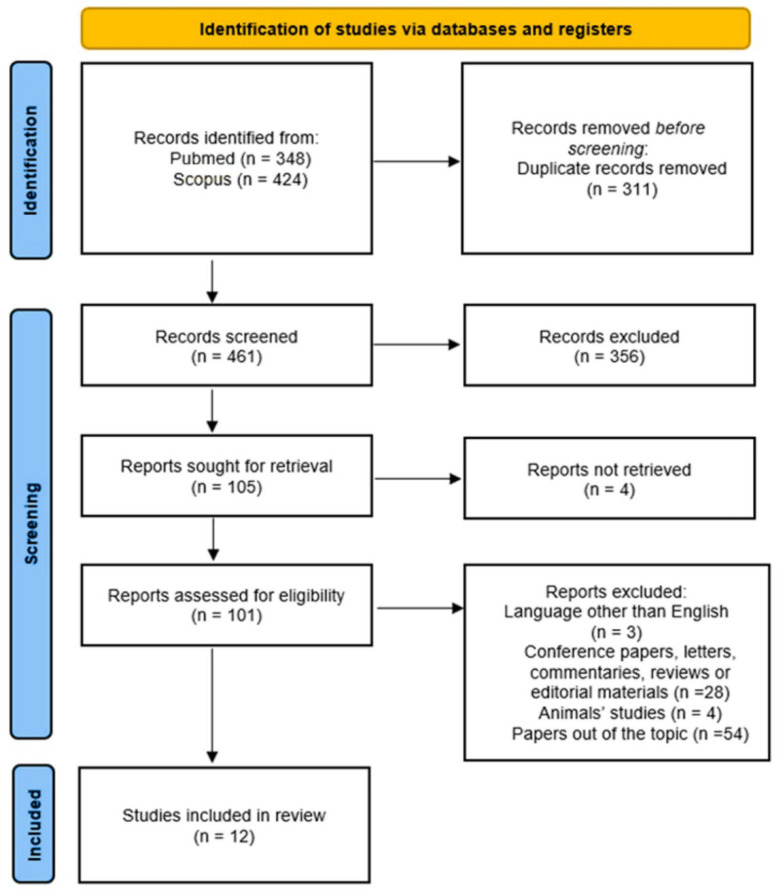
PRISMA flow diagram followed in this review.

We performed a systematic literature search according to the current Preferred Reporting Items for Systematic Reviews and Meta-Analyses Statement (PRISMA) criteria (Figure 1). We searched PubMed and Scopus databases for papers published between 1 January 1997 (note that the first paper on the genetic basis of BrS was published at the end of 1996) and 25 December 2021. We used a search string (restricted to the terms in the paper titles and abstracts) in which, using the Boolean operator “AND”, we combined the term “Brugada Syndrome” with the terms “fibrosis or scar or myocardial inflammation or structural heart disease or structural anomalies or structural abnormalities or histological anomalies or histological abnormalities or histological substrate or biopsies or fatty infiltration or ACM or ARVD or ARVC”. We developed and applied one search strategy for each database. Two authors independently performed a preliminary search and retrieved and selected articles that fulfilled the inclusion criteria: research studies written in English that evaluated a possible correlation between BrS and certain structural cardiac alterations (macroscopic/microscopic) and/or cardiomyopathies.

Our preliminary research identified 772 papers, 348 through PubMed and 424 through Scopus. After the removal of 311 duplicates, 356 papers were excluded as they did not meet the inclusion criteria based on the title and abstract analyses. Of the 105 articles remaining, 4 were excluded due to the unavailability of the full text. Hence, a total of 101 papers were assessed for eligibility. Full texts of reviews, case reports, experimental studies in animal models, conference articles, articles that did not focus on structural cardiac abnormalities in BrS, and articles that were not published in English were removed from the pool of eligible papers. Following the exclusion of all articles that did not meet our inclusion criteria, 12 eligible publications were included in our analysis and were critically reviewed by three investigators who extracted data relevant to the purpose of the present study. Selected studies are presented in two different paragraphs depending on the kinds of samples that were processed for histological analysis (samples collected from explanted heart or during autopsies versus endomyocardial biopsy samples). All authors agreed on the final data included in our study. Eligible papers were synthetized in a table, considering these variables: number of the reference, number of the cases and of the controls, kind of samples (endomyocardial biopsy vs explanted heart/autopsy samples), technique used for microscopic analysis, main microscopic findings, and whether genetic testing was performed. 

## 3. Results

### 3.1. Explanted Heart/Autopsy Samples

Two relevant case reports and two relevant case-series studies were identified. Assessment of formalin-fixed paraffin-embedded explanted heart tissue from a young individual with BrS and a clinical history of recurring VF [19], revealed moderate hypertrophy of the right ventricular wall (12 mm) and focal endocardial fibroelastosis. Moreover, in the RV (in the lateral wall and, especially, in the right ventricular outflow tract [RVOT]), significant fatty infiltration that reached the subendocardium was evident and was associated with interstitial fibrosis. The report excluded ACM because there was no evidence of transmural fatty infiltration, myocyte alterations, or inflammatory infiltrates (Table 1).

An autopsy of a 30-year-old victim of BrS [20] revealed biventricular contraction band necrosis and significant fatty tissue deposition in the RVOT. There were fewer cells of the sinus node, which was surrounded by fatty tissue and prominent fibrosis. Additionally, in two autopsy populations, including six autopsy-negative sudden deaths cases with (at least) a first-degree blood relative affected by BrS and six cases of non-cardiac deaths (as controls), individuals with BrS showed an increased amount of collagen [21]. The RVOT and epicardium demonstrated the greatest amount of fibrosis, and reduced expression of connexin-43 was observed in the RVOT. All hearts exhibited fibrosis, independent of the presence of *SCN5A* pathogenic variants. Together, these data suggested that in BrS, at the epicardial surface, interstitial fibrosis and reduced gap junction expression in the RVOT could lead to electrical anomalies. In further support of this finding, in 28 hearts from SCD cases with a non-confirmed diagnosis of BrS, the ventricular myocardium exhibited a higher proportion of collagen, irrespective of sampling location or myocardial layer (the highest proportion was found in the RVOT epicardium in individuals with suspected BrS) (Table 1) [22]. There was no statistically significant association reported between *SCN5A* genotype and histotype. 

### 3.2. Endomyocardial Biopsies

Two relevant case reports and six relevant case-series studies were identified. Explanted heart and autopsy samples showed the presence of fibrosis and collagen deposition and reduced expression of connexin-43, together potentially leading to electrical anomalies associated with BrS; however, endomyocardial biopsies exhibited inflammation and fatty infiltration (hallmarks of ACM). For example, a relationship between BrS and ACM was suggested, due to the observation of the fatty replacement of myocardium in a biopsy sample of an RV septum from a 73-old-year man with a history of syncopal episodes and precordial oppression, who was then diagnosed with BrS (Table 2) [23]. After identification of this possible association, biopsies in the septal-apical region of the LV and RV of 18 patients with BrS were performed [24]. From these samples, lymphocytic myocarditis (mainly activated T lymphocytes) associated with focal areas of myocyte necrosis in 14 cases was identified (myocarditis was biventricular in 6 cases, while in 8 cases inflammatory infiltrates were exclusively in the RV). Additionally, 4 cases showed evidence of viral genomes. The remaining 4 cases carried rare *SCN5A* variants, which are potentially associated with BrS (but not with a conclusive role) and presented abnormal levels of myocyte apoptosis. These data suggest a potential link between inflammation and BrS. Among biopsies collected from the RVOT areas of abnormal voltage identified under 3-dimensional electroanatomic mapping (3D-EAM) guidance from 30 BrS cases [25], 12 cases demonstrated myocardial inflammation with lymphomononuclear infiltrates, while 3 demonstrated an association between inflammatory infiltrates and myocyte necrosis (indicating an active myocarditis). All cases with abnormal structural findings also had interstitial and replacement fibrosis, as well as a statistically significant association between inflammation and inducibility with programmed ventricular stimulation (PVS)/extent of bipolar low voltage areas [25]. No statistically significant association between genotype and clinical/microscopic phenotype was reported. In stained myocardial samples obtained from one young case of SCD and from nine BrS patients, the expression of three proteins (α-cardiac actin, keratin-24, and connexin-43) and a sodium channel was assessed [26]. All cases exhibited abnormal aggregates of the three proteins and sodium channel within the sarcoplasm of the myocardium compared to healthy controls, suggesting that trafficking defects may be implicated in the pathogenesis of BrS. These findings were associated with the presence of antibodies against α-cardiac actin, α-skeletal actin, keratin, and connexin-43 in the sera of BrS patients, suggesting an autoimmune response. The authors stressed the relevance of connexin-43 anomalies, highlighting that in animal models, this protein is less abundant in the RVOT epicardium (Table 2). 

Fat deposition and oxidative stress may also trigger fibrosis and structural abnormalities that could potentially be associated with BrS. Endomyocardial biopsies from the septum (86%) and/or the RV/RVOT (76%) and/or the RV apex (57%) of 21 patients with a clinical BrS diagnosis showed no signs of acute inflammation [27]. However, approximately 50% of cases exhibited moderate cellular hypertrophy and fatty replacement of the myocardium, and less than one-fourth of cases had moderate fibrosis. In 4 patients in which there was predominant fatty replacement, criteria for ACM were not definitively met. Histotype and genotype were not correlated. The authors considered it unlikely that the reported findings could represent an arrhythmogenic origin. Biopsies at the junction between the septum and anterior RV free wall of a 65-year-old man with BrS demonstrated areas of fibro-fatty replacement covering 66% of the biopsy area [28]. The histomorphometric criteria for diagnosis of ACM were not definitively met. Additionally, biopsies on the upper septal region of the RV of 25 patients with a clinical diagnosis of BrS and inducible VF [29] showed moderate-to-severe fatty infiltration in 5 patients and showed myocyte degeneration (apoptotic zone), fibrosis, and lymphocyte infiltration in 4 patients. There was no detected correlation between clinical/electrophysiological phenotype and histotype, but a relationship between histological anomalies and slow conduction at the RVOT is possible. In patients with a documented history of VF, the 4-hydroxy-2-nonenal (HNE)-modified protein-positive area (a marker of lipid peroxidation and indicator of oxidative stress levels) was larger in endomyocardial biopsies from the RV side of the septum of 68 patients with a clinical diagnosis of BrS [30]. This finding was especially true if only patients without *SCN5A* variants were considered. Therefore, in individuals who do not carry *SCN5A* variants, oxidative stress could be involved in arrhythmogenesis, likely inactivating cardiac Na^+^ channels (Table 2).

### 3.3. Genetics

All manuscripts focused on structural alterations in BrS included a total of 209 cases. Genetic testing was performed in 161, and 36 cases carried a rare variant in the *SCN5A* gene (22.36%). This percentage is according to the widely accepted genetic yield in BrS [31], with *SCN5A* being the main gene currently associated with this arrhythmogenic syndrome [32]. Other minor genes encoding sodium subunits or associated proteins have been proposed as potential causes of BrS, but further studies should be conducted to conclude their definite role [3]. Due to some of the manuscripts being published more than five years ago, we performed an update following the American College of Medical Genetics and Genomics (ACMG) recommendations [33], according to our recent approach in the clinical translation of genetic diagnosis [31,34]. We identified only 16 cases (9.93%) who had a Likely Pathogenic (LP) or Pathogenic (P) variant explaining the genetic origin of BrS (Table 3). Most rare variants currently remain as VUS (Variant of Unknown Significance) due to the lack of enough conclusive data. Other cases diagnosed with BrS but without a positive *SCN5A* genetic diagnosis could be due to other genetic alterations in this gene [35] or in other genes [36]. However, it is also important to remark that only in 57 cases reported in the three most recent studies [22,25,26], a comprehensive genetic analysis including gene encoding cardiomyopathies were performed.

## 4. Discussion

BrS is currently classified as a purely electrical cardiac disease, but structural alterations identified in some cases suggest a potential reclassification of BrS as a cardiomyopathy. It is possible that dysfunctional ion channels lead to abnormal apoptosis and to a significant inflammatory/immune reaction and subsequent fibrosis in RV. An alternative hypothesis is that certain ion channel mutations result in altered excitation–contraction coupling causing cardiac remodeling [37]. Despite current arguments about this point, during the 30 years since first publication, none of the published cases with a definitive diagnosis of BrS have progressed to the definitive diagnosis of any cardiomyopathy during follow-up. Of all the analyzed manuscripts concerning structural alterations, few were performed by expert cardiopathologists, and this fact may represent a limitation due to the particular technical difficulty of microscopic diagnosis. Some centers included cardiac magnetic resonance (CMR) as part of BrS assessment despite not being included in current guidelines [32]. Therefore, further studies focused on analyzing potential correlations between BrS and structural abnormalities are needed to clarify whether BrS can definitively be reclassified as a cardiomyopathy.

Our comprehensive analysis identified recurring microscopic features of acute and chronic inflammation in the RVOT of BrS cases. Despite signs of acute inflammation, it is not a definitive hallmark of BrS but may trigger arrhythmias, especially in genetically predisposed hearts [25,26]. However, no conclusive studies have been published to date specifically examining this association, and the cause of myocardial inflammation remains undetermined. Increased collagen inside the myocardium represents a frequent feature of BrS and is predominant in the RV in both autopsy and endocardial biopsy samples [22]. However, this evidence is limited, as many of the patients studied did not have a confirmed clinical diagnosis of BrS. Another issue concerning fibrosis localization is the significance given to the collagen localized in the extraventricular parts of the conduction system [20]. The presence of collagen in this area is considered physiological; however, a significant amount of fibrosis can be abnormal, especially in young individuals [38]. For instance, an autopsy-negative case of sudden death in the young showed abnormal fibrosis of the sino-atrial node and the presence of a rare variant in the *SLMAP* gene (a minor gene potentially associated with BrS) [38]. In general, the presence of fibrosis in both the subendocardium and subepicardium has also been observed in other conditions (e.g., early repolarization syndrome) that are referred as “J-waves syndromes” and share the same arrhythmogenesis and the ECG changes of BrS [21,38].

As with inflammation, there is no clear evidence to date about fibrosis as a hallmark in BrS despite the presence of fibrosis in heart walls being widely accepted as proarrhythmogenic. Currently, data published identifies histological alterations in RV and in the RVOT of BrS patients. Transduction of electrical signals through myocytes is mainly due to connexin-43, and a reduction in this protein in the RVOT in BrS cases has been reported [21]; however, it is unclear if this phenomenon occurs before or after fibrosis. Therefore, further studies should seek to clarify if fibrosis identified in BrS samples could be a cause or consequence of arrhythmogenesis. In addition, electron beam computed tomography detected structural abnormalities on the RVOT and on the inferior wall of the RV that seem to be related to the onset of premature ventricular contractions and the initiation of VF [7]. RVOT is a critical part of the conduction system; thus, BrS may involve the abnormal expression of cardiac neural crest cells during embryonic myocardial development of the RVOT (whose characteristics differ from those of the surrounding myocardium) [39].

Recurrent histological features identified in BrS cases (myocardial fibrosis and the presence of inflammatory infiltrates) suggest a potential overlap between BrS and ACM histotypes. Debate persists surrounding whether BrS could be a cardiomyopathy or a phenotypic variant of ACM. Having the ability to make a differential diagnosis between BrS and ACM is crucial because in BrS cases that present ACM features, arrhythmic risk can be higher, and, in general, deciding the therapeutic strategy can be challenging [40,41]. This differential diagnosis is not always easier if clinical information is considered. For instance, patients with a definite diagnosis of ACM may exhibit an ECG pattern of BrS with a longer PQ interval and longer QRS duration, even if transient [42]. Therefore, in these cases, imaging data should also be considered. For instance, echocardiography can help in this differential diagnosis because BrS patients tend to have a mild alteration of RVOT morphology and motion but in the absence of overall dilation and dysfunction of the RV, typical of ACM [43,44]. Moreover, CMR can help to evaluate the fatty infiltration of the myocardium and the RV wall kinetics, helping in the differential diagnosis [45,46,47,48]. Data published to date state that early stages of ACM show alterations in ECG readings that are also observed in BrS cases, and discerning both entities is a challenge. Therefore, it cannot be ruled out that BrS and ACM share pathophysiological mechanisms and represent phenotypic and dynamic expressions of the same disease spectrum. Long-term follow-up is, therefore, required. Identification of the phenotype is important because of the clinical implications for both risk stratification and patient management. High-risk patients with a purely symptomatic electrical disorder may be more suitable for implantable cardioverter defibrillator (ICD). Whereas depending on the extent of the structural changes, selective ablation or drug therapy might be preferred. Future studies should focus on developing better standardized methods to differentiate BrS from ACM, to be evaluated by a multidisciplinary team of experts to ensure maximum diagnostic yield.

Improvement in genetic screening may help to clarify the diagnosis; however, it is not currently a viable solution, as the role of some potentially pathogenic variants has yet to be clarified, and a contribution of several genes in the development of the phenotype cannot be excluded. A comprehensive genetic analysis including all genes currently associated with BrS and ACM should be used in clinical or forensic settings [49]. However, even with genetic testing, differential diagnosis can be difficult because, for instance, rare variants located in the *PKP2* gene may be a potential cause of both ACM and BrS [12]. Yet, recent work identified one of these rare variants that was previously associated with BrS as a definitive cause in an ACM family [50]. Our group performed a comprehensive genetic interpretation of all rare variants in *PKP2* potentially associated with BrS, and none allowed a definite genotype–phenotype association [11]. In addition, less than 2% of ACM patients harbor rare *SCN5A* variants [51], but no conclusive role of these rare variants in ACM has been reported to date. These findings reinforce the necessity of further studies that include patients with a clear BrS diagnosis and ACM and a comprehensive genetic diagnosis, which would clarify the deleterious role of the identified rare variants. We recommend including a complete genotype–phenotype segregation in relatives to conclude a definitive genetic component, which could be translated into clinical practice.

### Limitations

We cannot definitively state that all manuscripts detailing structural alterations in BrS patients are included in our search using the PRISMA system at the time of our search. To assure a comprehensive search, we performed additional searches in Index Copernicus (www.en.indexcopernicus.com), Google Scholar (www.scholar.google.es), Springer Link (www.link.springer.com), Science Direct (www.sciencedirect.com), the Excerpta Medica Database (www.elsevier.com/solutions/embase-biomedical-research), and the IEEE Xplore Digital Library (www.ieeexplore.ieee.org/Xplore/home.jsp (all accessed on 25 December 2021)). After performing these additional searches, no other data was included. BrS patients who suddenly died or had a clinical indication for an ablation are high-risk patients and do not cover the entire spectrum of BrS patients.

## 5. Conclusions

BrS is currently considered a channelopathy; however, the identification of structural findings in some cases highlights the potential complex interplay between these structural alterations and ion channel dysfunction. The recurrence of some structural features in BrS should be carefully considered because in some of these cases there was an ECG pattern mimicking BrS (BrS phenocopies), but no definite diagnosis of BrS was reported. To overcome these issues, we recommend always performing a comprehensive investigation including all possible sources of information to select cases with a certain diagnosis of BrS. In addition, a close follow-up is strongly recommended, as throughout the 30 years since the first BrS publication, none of the published cases with a definitive diagnosis of BrS have progressed to the definitive diagnosis of any cardiomyopathy. Taking all data into account, we conclude that there is currently not enough evidence supporting a reclassification of BrS as a cardiomyopathy or an autoimmune disease. However, it should be noted that in autopsies, the observation of microscopic heart anomalies does not justify the exclusion of BrS as a possible diagnosis, so far. Therefore, new data may help to clarify the widely accepted classification of a “classic” channelopathy without structural heart alterations.

## Figures and Tables

**Table 1:** Summary of the literature review regarding explanted hearts/autopsy samples.

Reference	Cases	Controls	Samples	Technique for Microscopy	Main Findings	Genetic Testing
Coronel [19]	1	0	Explanted heart	Hematoxylin-eosin and picrosirius red	Hypertrophy of the right ventricular wall, focal endocardial fibroelastosis, fatty infiltration, interstitial fibrosis	Yes
Moritomo [20]	1	0	Autopsy samples	Hematoxylin-eosin, Masson’s trichrome and Azan Mallory	Reduction number of node cells and increased fatty tissue and fibrosis in the sinus node	No
Nademanee [21]	6	6	Autopsy samples	Hematoxylin-eosin and elastic Van Gieson and connexin-43 immunofluorescent	An increased collagen and fibrosis, (RVOT), reduction in connexin-43 signal	Yes
Miles [22]	28	29	Autopsy samples	Hematoxylin-eosin and picrosirius red	Increased collagen content in both ventricles, especially in RVOT epicardium	Yes

**Table 2:** Summary of the literature review regarding biopsies.

Reference	Cases	Controls	Samples	Technique for Microscopy	Main Findings	Genetic Testing
Izumi [23]	1	0	Biopsy	Hematoxylin-eosin	Fatty replacement of myocardium	No
Frustaci [24]	18	0	Biopsies	Hematoxylin-eosin, Miller’s elastic Van Gieson, and Masson’s trichrome	Lymphocytic myocarditis with focal areas of myocytes necrosis, hypertrophy, and diffuse vacuolization of cardiomyocytes with cytoplasm degeneration	Yes
Zumhagen [27]	21	12	Biopsies	Hematoxylin-eosin and Miller’s elastic Van Gieson	Moderate hypertrophy and fatty replacement of the myocardium, moderate fibrosis	Yes
Marras [28]	1	0	Biopsy	Masson’s trichrome	Fibro-fatty replacement and mild endocardial fibrous thickening	No
Ohkubo [29]	25	0	Biopsies	Hematoxylin-eosin	Moderate-to-severe fatty infiltration/myocyte degeneration, arrangement disorder, interstitial fibrosis, and lymphocyte infiltration	No
Tanaka [30]	68	0	Biopsies	Hematoxylin-eosin, Masson’s trichrome, and immunohistochemical CD45, CD68, 4-hydroxy-2-nonenal-modified protein	Large 4-hydroxy-2-nonenal-modified protein areas in those without *SCN5A* mutation and with history of ventricular fibrillation	Yes
Pieroni [25]	30	0	Biopsies	Hematoxylin-eosin, Masson’s trichrome, and immunohistochemistry anti-CD45RO	Myocardial inflammation with lymphomononuclear infiltrates	Yes
Chatterejee [26]	9	1	Biopsies	Immunohistochemistry	Abnormal myocardial expression of alfa-cardiac actin, alfa-skeletal actin, keratin-24, connexin-43, Nav1.5	Yes

**Table 3:** Genetic data of variants in the *SCN5A* gene.

Publication	Zone	Region	Nucleotide	Protein	dbSNP/ClinVar	GnomAD (MAF)	ACMG 2022	Genes Analysed
Coronel et al. 2005 [19]	C-Terminal	Intracellular	c.5803G>A	p.Gly1935Ser	rs199473637/VUS	7/248912 (0.0028%)	VUS	*PKP2, DSP, RyR2*
Zumhagen et al. 2008 [27]	S5 (DII)	Pore	c.2582_2583del	p.Phe861TrpfsTer90	rs794728914/P	NA	P	No
Zumhagen et al. 2008 [27]	Loop DII-DIII	Intracellular	NA	p.Pro1002HisfsTer25	NA	NA	VUS	No
Zumhagen et al. 2008 [27]	Loop DIII-DIV	Intracellular	c.4477_4479del	p.Lys1493del	rs869025522/LP	1/151978 (0.0006%)	LP	No
Zumhagen et al. 2008 [27]	S2 (DIV)	Voltage Sensor	c.4720G>A	p.Glu1574Lys	rs199473620/VUS	NA	VUS	No
Zumhagen et al. 2008 [27]	S6 (DIV)	Pore	c.5290G>T	p.Val1764Phe	rs199473309/NA	NA	VUS	No
Zumhagen et al. 2008 [27]	C-Terminal	Intracellular	c.5435C>A	p.Ser1812Ter	rs371891414/LP	NA	LP	No
Frustaci et al. 2009 [24]	Loop S5-S6 (DI)	Extracellular	c.1127G>A	p.Arg376His	rs199473101/LP	2/247596 (0.0008%)	LP	*PKP2, RyR2*
Frustaci et al. 2009 [24]	Loop DII-DIII	Intracellular	c.3068G>A	p.Arg1023His	rs199473592/VUS	70/247778 (0.0283%)	LB	*PKP2, RyR2*
Frustaci et al. 2009 [24]	S4 (DIV)	Voltage Sensor	c.4930C>T	p.Arg1644Cys	rs199473287/P	1/251472 (0.0003%)	P	*PKP2, RyR2*
Frustaci et al. 2009 [24]	C-Terminal	Intracellular	c.5903T>G	p.Ile1968Ser	rs199473639/VUS	4/244136 (0.0016%)	VUS	*PKP2, RyR2*
Nademanee et al. 2015 [21]	Loop DI-DII	Intracellular	c.1582A>T	p.Ser528Cys	NA	NA	VUS	No
Nademanee et al. 2015 [21]	S5 (DII)	Pore	c.2537T>G	p.Leu846Arg	NA	NA	VUS	No
Nademanee et al. 2015 [21]	S6 (DIII)	Pore	c.4385T>A	p.Leu1462Gln	NA	NA	VUS	No
Pieroni et al. 2018 [25]	S6 (DII)	Pore	c.2798T>C	p.Leu933Pro	NA	NA	VUS	147 genes (panel)
Pieroni et al. 2018 [25]	Loop (S5-S6) DIV	Extracellular	c.5102T>G	p.Met1701Arg	NA	NA	VUS	147 genes (panel)
Pieroni et al. 2018 [25]	Loop S5-S6 (DIII)	Extracellular	c.4300_4311del	p.Tyr1434_Gln1437del	NA	NA	LP	147 genes (panel)
Pieroni et al. 2018 [25]	S2 (DIV)	Voltage Sensor	c.4720G>A	p.Glu1574Lys	rs199473620/VUS	NA	VUS	147 genes (panel)
Pieroni et al. 2018 [25]	S4 (DIV)	Voltage Sensor	c.4930C>T	p.Arg1644Cys	rs199473287/P	1/251472 (0.0003%)	P	147 genes (panel)
Pieroni et al. 2018 [25]	Loop DII-DIII	Intracellular	c.3352C>T	p.Gln1118Ter	rs869025520/P	NA	P	147 genes (panel)
Chatterjee et al. 2020 [26]	Loop S5-S6 (DI)	Extracellular	c.1007C>T	p. Pro336Leu	rs199473093/VUS	NA	LP	Gene panel
Chatterjee et al. 2020 [26]	Loop DII-DIII	Intracellular	c.3352C>T	p.Gln1118Ter	rs869025520/P	NA	P	Gene panel
Chatterjee et al. 2020 [26]	Loop S5-S6 (DI)	Extracellular	c.844C>G	p.Arg282Gly	rs199473082/VUS	NA	VUS	Gene panel
Chatterjee et al. 2020 [26]	NA	NA	c.3508+1G>A	NA	NA	NA	VUS	Gene panel
Chatterjee et al. 2020 [26]	Loop DIII-DIV	Intracellular	c.4501C>G	p.Leu1501Val	rs199473266/VUS	5/251446 (0.0019%)	VUS	Gene panel
Chatterjee et al. 2020 [26]	S6 (DIII)	Pore	c.4387A>T	p.Asn1463Tyr	rs199473614/VUS	NA	VUS	Gene panel
Chatterjee et al. 2020 [26]	Loop DIII-DIV	Intracellular	c.4477_4479del	p.Lys1493del	rs869025522/LP	1/151978 (0.0006%)	LP	Gene panel
Chatterjee et al. 2020 [26]	Loop S5-S6 (DI)	Extracellular	c.1127G>A	p.Arg376His	rs199473101/LP	2/247596 (0.0008%)	LP	Gene panel
Chatterjee et al. 2020 [26]	Loop S5-S6 (DIV)	Extracellular	c.5027T>C	p.Met1676Thr	rs750013499/LP	1/251494 (0.0003%)	LP	Gene panel
Chatterjee et al. 2020 [26]	Loop S1-S2 (DIII)	Extracellular	c.3695G>A	p.Arg1232Trp	rs199473206/VUS	6/250110 (0.0023%)	VUS	Gene panel
Miles et al. 2021 [22]	Loop S3-S4 (DIII)	Intracellular	c.3944C>G	p.Ser1315Ter	rs1261656894/NA	NA	LP	174 genes (panel)
Miles et al. 2021 [22]	N-Terminal	Intracellular	c.50C>T	p.Thr17Ile	NA	NA	VUS	174 genes (panel)
Miles et al. 2021 [22]	Loop S5-S6 (DIV)	Extracellular	c.5038G>A	p.Ala1680Thr	rs199473294/VUS	10/251494 (0.0039%)	VUS	174 genes (panel)
Miles et al. 2021 [22]	S5 (DI)	Pore	c.673C>T	p.Arg225Trp	rs199473072/LP	3/242066 (0.0012%)	LP	174 genes (panel)
Miles et al. 2021 [22]	S1 (DIII)	Voltage Sensor	c.3665T>G	p.Leu1222Arg	NA	NA	VUS	174 genes (panel)
Miles et al. 2021 [22]	S3 (DIV)	Voltage Sensor	c.4850_4852delTCT	p.Phe1617del	rs749697698/LP	5/250930 (0.0019%)	LP	174 genes (panel)

dbSNP, database single nucleotide polymorphism; MAF, Minor Allele Frequency; ACMG, American College of Molecular Genetics; VUS, Variant of Unknown Significance; P, Pathogenic.

## Data Availability

Not applicable.
